# Use of non-invasive respiratory supports in high-intensity internal medicine setting during the first two waves of the COVID-19 pandemic emergency in Italy: a multicenter, real-life experience

**DOI:** 10.1007/s11739-023-03371-z

**Published:** 2023-07-20

**Authors:** Ernesto Crisafulli, Giulia Sartori, Alice Vianello, Alessio Maroccia, Elisa Lepori, Massimiliano Quici, Chiara Cogliati, Massimo Salvetti, Anna Paini, Carlo Aggiusti, Fabio Bertacchini, Fabiana Busti, Giacomo Marchi, Maria Lorenza Muiesan, Domenico Girelli

**Affiliations:** 1 Department of Medicine, Respiratory Medicine Unit and Section of Internal Medicine, University of Verona and Azienda Ospedaliera Universitaria Integrata of Verona, Largo L. A. Scuro, 10, 37124 Verona, Italy; 2 Department of Medicine, Section of Internal Medicine, University of Verona and Azienda Ospedaliera Universitaria Integrata of Verona, Verona, Italy; 3 Internal Medicine, L.Sacco Hospital, ASST-FBF-Sacco, Milan, Italy; 4 Department of Biochemical and Clinical Sciences, University of Milan, Milan, Italy; 5 Dipartimento Di Scienze Cliniche E Sperimentali, Università Di Brescia, Brescia, Italy; 6 SSVD Medicina Di Urgenza ASST Spedali Civili Brescia, Brescia, Italy; 7 UOC 2° Medicina Generale ASST Spedali Civili Brescia, Brescia, Italy

**Keywords:** COVID-19, Non-invasive respiratory supports, Acute respiratory failure, Internal medicine high-dependency wards, Do-not-intubate, Outcomes

## Abstract

During the first two waves of the COVID-19 emergency in Italy, internal medicine high-dependency wards (HDW) have been organized to manage patients with acute respiratory failure (ARF). There is heterogeneous evidence about the feasibility and outcomes of non-invasive respiratory supports (NIRS) in settings outside the intensive care unit (ICU), including in patients deemed not eligible for intubation (i.e., with do-not-intubate, DNI status). Few data are available about the different NIRS modalities applied to ARF patients in the newly assembled internal medicine HDW. The main aim of our study was to describe a real-life experience in this setting of cure, focusing on feasibility and outcomes. We retrospectively collected data from COVID-19 patients with ARF needing NIRS and admitted to internal medicine HDW. Patients were treated with different modalities, that is high-flow nasal cannula (HFNC), continuous positive airway pressure (CPAP), or non-invasive mechanical ventilation (NIMV). Switching among different NIRS during the hospitalization and the success rate (weaning with the same NIRS) or failure (endotracheal intubation-ETI or in-hospital death) were recorded. Three hundred thirty four ARF patients (median age 74 years), of which 158 (54%) had a DNI status, were included. CPAP, NIMV, and HFNC's success rates were 54, 33, and 13%, respectively. Although DNI status was strongly associated with death (Gehan–Breslow–Wilcoxon test *p* < 0.001), an acceptable success rate was observed in these patients using CPAP (47%). Multivariate regression models showed older age (odds ratio—OR 4.74), chronic ischemic heart disease (OR 2.76), high respiratory rate after 24 h (OR 7.13), and suspected acute respiratory distress syndrome—ARDS (OR 21.1) as predictors of mortality risk or ETI. Our real-life experience shows that NIRS was feasible in internal medicine HDW with an acceptable success rate. Although DNI patients had a worse prognosis, the use of NIRS represented a reasonable chance of treatment.

**Supplementary Information:**

The online version contains supplementary material available at 10.1007/s11739-023-03371-z.

## Introduction

In Italy, the coronavirus disease 2019 (COVID-19) pandemic will be remembered for the unexpected outbreak in the northern part of the country with a broad and rapid diffusion [1], resulting in massive hospital admission of patients with severe disease [2]. Indeed, COVID-19 patients frequently developed pneumonia with hypoxemic acute respiratory failure (ARF) requiring progressive respiratory support [2, 3], from nasal cannula to non-invasive respiratory supports (NIRS). However, some questions remain about the utility, safety, and outcome of NIRS strategies [3, 4] represented by the high-flow nasal cannula (HFNC), continuous positive airway pressure (CPAP), and non-invasive mechanical ventilation (NIMV). An extensive collection of data evaluating different NIRS strategies [4] allows focus on the possibility of treatment escalation (the endotracheal intubation-ETI or intensive care unit-ICU admission) and to evaluate the impact on outcomes [4].

In Italy, although intensive care beds were more than doubled during the first waves of the pandemic, their occupancy was constantly close to 100% due to the overwhelming number of patients with ARF, making necessary to adopt emergency strategies for ventilatory support outside the ICU [1]. This was proven feasible to cope with the massive demand for ventilatory assistance [5, 6]. Available evidence suggests that in candidates to full treatment escalation, 37% progressed to invasive mechanical ventilation (IMV) with a survival rate of 78% [4]. On the other hand, the survival rate was very low (30%) among the patients for whom NIRS was a priori judged as the ceiling of treatment [4]. Such patients, generally referred as do-not-intubate (DNI) subgroup, were reported to represent up to nearly 40% of total admissions [7] and a particularly challenging category in different observational studies [6, 7]. Nevertheless, a marked heterogeneity exists between available studies in patient populations, including age, comorbidities, COVID-19 severity at baseline, ward settings, and techniques used [4]. In a recent meta-analysis, the overall intra-hospital mortality of patients receiving NIRS outside the ICU was 36% [8]. Of note, most COVID-19 patients in Italy have been admitted to internal medicine units [9], not rarely including newly created high-dependency wards (HDW) with NIRS facilities. In such settings, where elderly patients with multiple comorbidities and DNI status were frequently admitted, some positive outcomes have been reported [10–12].

To better evaluate the feasibility and effectiveness of NIRS in internal medicine HDW with a particular focus on patients with a DNI status, we analyzed data from three centers from northern Italy during the first two waves of the COVID pandemic. Since different types of NIRS and interfaces were used, data were stratified accordingly, and shifts among different supports were also described and analyzed.

## Methods

### Study cohort and setting

We retrospectively considered all consecutive patients with confirmed COVID-19 infection with pneumonia causing ARF, admitted to three dedicated internal medicine HDW in northern Italy (at the University Hospitals of Verona, Brescia, and Milano) from March 2020 to May 2020 and from October 2020 to March 2021, corresponding to the first two pandemic waves in Italy. In each HDW, all staff components have been specifically trained, and a pneumologist has instructed any technical aspect related to NIRS in the early training phase. The presence of ARF was defined according to the partial pressure of arterial oxygen to the fraction of inspired oxygen (PaO_2_/FiO_2_) ≤ 300, with or without respiratory distress needing a NIRS. We defined suspected ARDS (acute respiratory distress syndrome) as a clinical condition with a rapid worsening of PaO_2_/FiO_2_, the need for an increase in required pressure in the NIMV and CPAP treatment and the presence of progressive bilateral opacities at least on chest radiograph [13]. In fact, due to organizational issues, the radiological confirmation by the computed tomography (CT) scan, in our clinical context, has not been performed in all enrolled patients. Cardiac failure in patients with suspected ARDS has been evaluated by an objective assessment (echocardiography). The Hospital’s Ethics Committee approved the study protocol, conducted following Good Clinical Practices and the declarations of Helsinki.

### Measurements

Data related to anthropometric characteristics, time from onset of symptoms to admission, smoking habit, the prevalence of comorbidities, Sequential Organ Failure Assessment (SOFA) score, blood gas analysis, laboratory variables, and heart and respiratory rate were collected at admission. The pharmacological COVID-19 treatments were also collected. An intensivist of the local medical emergency team (MET) established a DNI status at admission, along with the floor internist, the patient themselves, and their relatives. Elements considered in the decision were the presence of cofactors, including advanced age and multiple comorbidities, ARF severity, and expected short-term death independently of COVID-19, overall predicting the futility of more aggressive approaches. In patients with no clear DNI status, a wait-and-see approach was adopted, with patients regularly screened (at least daily) by the MET plus on demand of the floor internist for possible ICU admission, reserving the possibility of a late ETI. The blood gas analysis assessment and the heart and respiratory rates were repeated 24 h after admission.

### Outcomes and complications

The primary outcome was the rate of success (weaning from the NIRS) or failure (need for ETI or in-hospital mortality). In addition, the clinical progression (secondary outcomes) was assessed according to the length of hospital stay (LHS) or the development of complications, with particular reference to suspected ARDS, shock, acute ischemic heart or arrhythmias, pulmonary embolism, bacteremia, and acute kidney or neurological impairments. Technical characteristics of NIRS, such as the interfaces used, pressure support (PS), positive end-expiratory pressure (PEEP), data related to high-flow nasal cannula (HFNC), the fraction of inspired oxygen (FiO_2_), and the time of NIRS use, were also collected.

### Statistical analysis

Data were reported as numbers (percentages of patients) for categorical variables or medians (1st quartile; 3rd quartile) for continuous variables due to the non-normal distribution, evaluated by the preliminary test of normality of Shapiro–Wilk. Categorical variables were compared using the chi-square test or the Fisher exact test, while continuous variables were assessed with the non-parametric Kruskal–Wallis H or Mann–Whitney tests, as appropriate.

Univariate and multivariate logistic regression analyses were performed to identify variables predictive of ETI or death (dependent variable). The independent variables tested in the univariate analyses were: age (≥ 75 years), sex, smoking habit, time from onset of symptoms to admission (+1 day), Glasgow score (+1 point), SOFA score (+1 point), comorbidities (presence of arterial hypertension, atrial fibrillation, chronic ischemic heart disease, diabetes mellitus, active cancer, cerebrovascular disease, kidney disease, chronic obstructive pulmonary disease, and asthma), laboratory data (hemoglobin, leukocytes, neutrophils, lymphocytes, C-reactive protein, procalcitonin, D-dimer, platelets, fibrinogen, lactate dehydrogenase-LDH), respiratory supports (CPAP considered as reference variable), all intercurrent complications and treatment used during the hospitalization (use of lopinavir/ritonavir, hydroxychloroquine, tocilizumab, remdesivir, systemic steroids, antibiotics, prophylactic or therapeutic dosage of low molecular weight heparin-LMWH). Moreover, in the model, the following variables have been evaluated at admission and 24 h: pH, arterial partial carbon dioxide pressure-paCO_2_, the ratio of the partial pressure of arterial oxygen to the fraction of inspired oxygen-PaO_2_/FiO_2_ (≤ 200), lactate (+1 mmol/L), respiratory (≥ 30 bpm), and heart rate (≥ 100 bpm). Variables that showed an association with *p* < 0.1 were included in the corresponding multivariate regression stepwise model. Strongly correlated variables (*r* >|± 0.3|) were excluded from the multivariate analyses. The multivariate model including 12 variables was adjusted for the HDW admitting the patient, sex, and NIRS. We then calculated the odds ratios (ORs) and 95% confidence intervals (CI) and the calibration ability with the Hosmer–Lemeshow goodness-of-fit test [14].

A sub-analysis has been performed on patients with a DNI status at admission. We evaluated mortality as a time-to-event variable and analyzed it using Kaplan–Meier survival curves. The Gehan–Breslow–Wilcoxon test was applied because it emphasizes early differences [15].

All statistical analyses were performed using IBM SPSS Statistics 24.0 (Armonk, New York, USA). A value of *p* < 0.05 was considered statistically significant.

## Results

A total of 334 consecutive patients with ARF related to COVID-19 were admitted during the study period to the internal medicine wards in Verona (*n* = 111, 33%), Brescia (*n* = 171, 51%), and Milano (*n* = 52, 16%), and treated with NIRS since admission. They were older patients (median age 74 years, 31% had an age over 80 years) with a high burden of comorbidity (50% had at least two comorbidities reported). They were stratified according to the different types of NIRS initially chosen, which was CPAP (55%), NIMV (38%), and HFNC (7%). Of note, 158 patients (54% of the total) were classified as DNI. As compared to CPAP, patients treated with NIMV since admission were older, more frequently current or former smokers, more hypoxemic with a higher respiratory rate and lactate level; they also had higher levels of leukocytes, neutrophils, procalcitonin, d-dimer, and fibrinogen, as well as higher use of antibiotics and LMWH at therapeutic dose. On the other hand, HFNC patients (in comparison to NIMV) were less frequently smokers, had lower respiratory rate and levels of leukocytes, neutrophils, and platelets. Table 1 reports the general characteristics of the patients considered.

**Table 1:** General characteristics of patients reported according to the respiratory support used at admission to the HDW

Variables	All patients *n* = 334	CPAP *n* = 183	NIMV *n* = 128	HFNC *n* = 23
Age, years	74 [62–81]	71 [58.7–80.2]	76 [67.2–80] *	79 [67–84]
Female, *n* (%)	112 (33)	61 (33)	43 (34)	8 (35)
Patients do-not-intubate, *n* (%)	158 (54)	88 (56)	59 (49)	11 (58)
Time from onset of symptoms to admission, days	6 [3–9]	6.5 [3–9.2]	7 [3–9]	5 [3–7]
Smoking habit, current or former, *n* (%)	76 (36)	39 (31)	35 (47) *	2 (17) §
Arterial hypertension, *n* (%)	206 (62)	119 (65)	74 (58)	13 (56)
Atrial fibrillation, *n* (%)	36 (11)	20 (11)	11 (8.6)	5 (22)
Chronic ischemic heart disease, *n* (%)	59 (18)	32 (17)	22 (17)	5 (22)
Diabetes mellitus, *n* (%)	76 (23)	42 (23)	29 (23)	5 (22)
Active cancer, *n* (%)	23 (6.9)	13 (7.1)	9 (7)	1 (4.3)
Cerebrovascular disease, *n* (%)	35 (10)	17 (9.3)	14 (11)	4 (17)
Kidney disease, *n* (%)	42 (13)	19 (10)	17 (13)	6 (26)*
COPD, *n* (%)	25 (7.5)	10 (5.5)	13 (10.2)	2 (8.7)
Asthma, *n* (%)	7 (2.1)	4 (2.2)	2 (1.6)	1 (4.3)
SOFA, score	2 [2, 3]	2 [2, 3]	2 [2, 3]	2 [2–4]
pH	7.47 [7.43–7.50]	7.47 [7.44–7.50]	7.46 [7.43–7.49]	7.48 [7.43–7.52]
PaCO_2_, mmHg	32.15 [30–36]	32 [29.6–36]	33 [30–37]	33 [31–36.5]
PaO_2_/FiO_2_	226.2 [168.8–279]	242.8 [171.4–292.4]	205.7 [149.7–252.4] *	213.8 [171.5–264.9]
Lactate, mmol/L	1.48 [1.06–2]	1.4 [1–1.8]	1.8 [1.15–2.35] *	1.33 [1–2.1]
Respiratory rate, bpm	24 [20–30]	24.5 [18–30]	26 [21–32.7] *	20 [18–23] §
Heart rate, bpm	88 [75–100]	88 [75–100]	88 [75–100]	82 [70–98]
Hemoglobin, g/dL	13.3 [12.1–14.3]	13.3 [12.2–14.4]	13.5 [12.2–14.2]	12.7 [11.2–13.1]
Leukocytes, 10^9^ cells/L	8.01 [5.43–10.78]	7.36 [5.15–9.75]	9.27 [6.73–12.93]**	7.1 [4.13–8.9]^§^
Neutrophils, 10^9^ cells/L	6.28 [4.05–9.06]	5.59 [3.77–8.48]	7.38 [4.79–10.5]**	4.7 [3.32–7.69]^§^
Lymphocytes, 10^9^ cells/L	8.3 [5.9–11.3]	7.59 [5.67–11.5]	8.80 [5.9–11.4]	8.95 [6.25–9.95]
C-reactive protein, mg/L	96 [51–159.1]	85.6 [42.7–146.3]	110 [59.1–173.2]	96.3 [56.7–140.8]
Procalcitonin, ng/mL	0.15 [0.10–0.40]	0.11 [0.10–0.24]	0.21 [0.10–0.64] *	0.11 [0.10–0.61]
D-dimer, ng/mL	1072.5 [612.5–2004.2]	818 [511.2–1733.2]	1359 [865.7–2289.2] **	1137 [712–2388.2]
Platelets, 10^9^/L	200 [153.7–264.2]	195 [156–259]	215 [160.5–282]	158 [135–249] §
Fibrinogen, mg/dL	600 [467–716]	579 [392–694]	632 [516.5–727] *	586 [443–631]
LDH, U/L	387 [300–482]	378 [306–465.2]	397.5 [290.7–484]	426 [305.5–547]
Lopinavir/Ritonavir, *n* (%)	23 (6.9)	14 (7.7)	9 (7)	0 (0)
Hydroxychloroquine, *n* (%)	41 (12)	28 (15)	13 (10)	0 (0)*
Tocilizumab, *n* (%)	7 (2.1)	4 (2.2)	3 (2.3)	0 (0)
Remdesivir, *n* (%)	29 (8.7)	11 (6)	14 (11)	4 (17)
Systemic steroids, *n* (%)	329 (98)	181 (99)	125 (98)	23 (100)
Antibiotics, *n* (%)	298 (89)	154 (84)	123 (96)*	21 (91)
Prophylactic LMWH, *n* (%)	207 (62)	127 (69)	66 (52)*	14 (61)
Therapeutic LMWH, *n* (%)	112 (33)	49 (27)	56 (44)*	7 (30)

Data are shown as median [25–75° percentiles] or number (percentages). Percentages are calculated on non-missing data

*CPAP* continuous positive airway pressure, *NIMV* non-invasive mechanical ventilation, *HFNC* high-flow nasal cannula, *SOFA* sequential organ failure assessment, *COPD* chronic obstructive pulmonary disease, *PaCO*_*2*_ arterial partial carbon dioxide pressure, *PaO*_*2*_*/FiO*_*2*_ the ratio of the partial pressure of arterial oxygen to the fraction of inspired oxygen, *LDH* lactate dehydrogenase, *LMWH* low molecular weight heparin

* *p* < 0.05 vs CPAP; ** *p* < 0.001 *vs* CPAP; § *p* < 0.05 vs NIMV

After 24 h of treatment with NIRS, the pre-to-post change (Δ) of gas analysis variables showed only a significant difference in lactate level in the HFNC group, which increased in comparison to patients using NIMV and CPAP; similarly, the respiratory rate was higher in HFNC as compared with NIMV (Table 2).

**Table 2:** Early impact of NIRS

Variables	All patients	CPAP	NIMV	HFNC
Δ pH	−0.01 [−0.05 to 0.02]	−0.01 [−0.04 to 0.023]	−0.02 [−0.05 to 0.025]	−0.03 [−0.06 to 0.005]
Δ PaCO_2_, mmHg	5 [0.85–9]	5 [0–8]	5 [1.5–9]	4.9 [1.57–10.75]
Δ PaO_2_/FiO_2_	−75.5 [−146.2 to 11.9]	−79.9 [−151.2 to 8.2]	−74.3 [−136.9 to 13.3]	−100.8 [−148.1 to 41.9]
Δ Lactate, mmol/L,	0 [−0.57 to 0.37]	0 [−0.48 to 0.29]	0 [−0.90 to 0.38]	0.7 [−0.05 to 1.12] * §
Δ Respiratory rate, bpm	−2 [−8 to 4]	−1 [−6.5 to 5]	−3 [−9.5 to 2]	3 [−5 to 7] §
Δ Heart rate, bpm	−12 [−25 to 0]	−14.5 [−27.7 to 0]	−10 [−21.5 to 1]	−10 [−22 to 2]

Data are shown as median [25–75° percentiles] of pre-to-post change (Δ) from the beginning of the respiratory support use (admission) and 24 h

*NIRS* non-invasive mechanical ventilation, *CPAP* continuous positive airway pressure, *NIMV* non-invasive mechanical ventilation, *HFNC* high-flow nasal cannula, *PaCO*_*2*_ arterial partial carbon dioxide pressure, *PaO*_*2*_*/FiO*_*2*_ the ratio of the partial pressure of arterial oxygen to the fraction of inspired oxygen

* *p* < 0.05 vs CPAP; § *p* < 0.05 vs NIMV

The evaluation of outcomes and complications during hospitalization is shown in Table 3. Compared to the CPAP group, the NIMV group included patients with more frequent need of ETI, intra-hospital mortality, and documented bacteremia (*p* < 0.001). No other significant differences were observed between the three study groups.

**Table 3:** Outcomes and complications

Variables	All patients	CPAP	NIMV	HFNC
Length of stay, days	16 [10–24]	15.5 [10.75–23]	15.5 [9–27]	16 [13–28]
Need for ETI/deaths, *n* (%)	150 (45)	63 (34)	77 (60) **	10 (43)
Suspected ARDS, *n* (%)	182 (54)	104 (57)	67 (52)	11 (48)
Shock, *n* (%)	22 (6.6)	10 (5.5)	10 (7.8)	2 (8.7)
Acute ischemic heart complication, *n* (%)	8 (2.4)	5 (2.7)	3 (2.3)	0 (0)
Acute arrhythmia, *n* (%)	28 (8.4)	18 (9.8)	8 (6.3)	2 (8.7)
Pulmonary embolism, *n* (%)	31 (9.3)	17 (9.3)	12 (9.4)	2 (8.7)
Bacteremia, *n* (%)	69 (21)	26 (14)	38 (30) *	5 (22)
Acute kidney complication, *n* (%)	41 (12)	18 (9.8)	19 (14.8)	4 (17.4)
Acute neurological complication, *n* (%)	26 (7.8)	14 (7.7)	9 (7)	3 (13)

Data are shown as median [25–75° percentiles] and number (percentages). Percentages are calculated on non-missing data

*CPAP* continuous positive airway pressure, *NIMV* non-invasive mechanical ventilation, *HFNC* high-flow nasal cannula, *ICU* intensive care unit, *PaCO*_*2*_ arterial partial carbon dioxide pressure, *PaO*_*2*_*/FiO*_*2*_ the ratio of the partial pressure of arterial oxygen to the fraction of inspired oxygen, *ARDS* acute respiratory distress syndrome

* *p* < 0.05 vs CPAP; ** *p* < 0.001 vs CPAP

Concerning the technical characteristics related to the first use of NIRS (interfaces), patients in helmets were supported prevalently with CPAP, while patients with total face mask by NIMV; there were few differences between CPAP and NIMV regarding the use of the oro-nasal mask. The PEEPs and the FiO_2_ were similar between CPAP and NIMV groups. The flow used in HFNC was 60 L/m. The time of treatments was very short in HFNC (shift to other NIRS after a median of 1 day) as compared to CPAP (*p* < 0.001) and NIMV (*p* < 0.001), while NIMV was used for more extended periods as compared to CPAP (median duration 6 and 3 days, respectively). Other technical aspects of the different NIRS are reported in Supplementary Information Table 1.

**Fig. 1:**
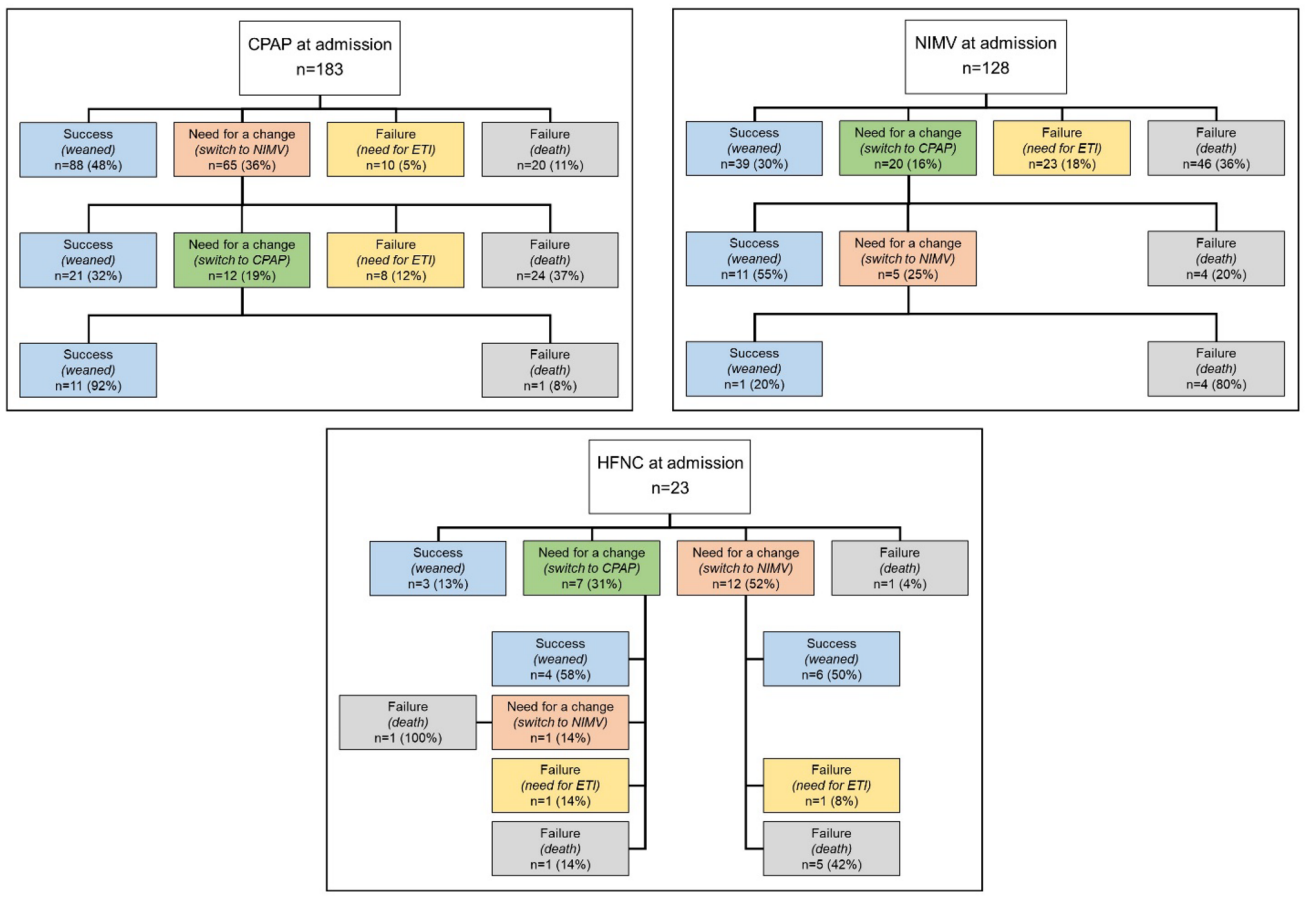
Flow diagram concerning the use of NIRS. *Abbreviations:* NIRS define non-invasive respiratory support; CPAP, continuous positive airway pressure; NIMV, non-invasive mechanical ventilation; HFNC, high-flow nasal cannula; ETI, endotracheal intubation

Figure 1 illustrates flow diagrams regarding the use of the three NIRS approaches during hospitalization, including the switches among different supports and the major outcomes (successful weaning, ETI, and death). Considering the first support since admission, CPAP showed a higher success rate (48%), followed by NIMV (30%) and HFNC (13%). In our experience, CPAP was superior to other NIRS, also considering the overall success rate, while weaning as a second approach (e.g., after switching from another treatment) was similar between CPAP and NIMV subgroups (12 and 14%, respectively). No patients used HFNC as a switch treatment after an initial failure. In general, the treatment switch was higher in CPAP patients (36% switch to NIMV), while only 16% of patients using NIMV switched to CPAP during hospitalization; 83% of patients using HFNC at admission switched to CPAP or NIMV. The failure of NIRS treatment (need for ETI and death) at any time was higher in patients using NIMV (54%) as compared to CPAP (16%).

Table 4 shows the univariate and multivariate models predicting a composite endpoint of ETI or in-hospital death. Concerning the use of NIRS and considering CPAP as a reference variable, only NIMV increased the probability of having ETI/death (OR 2.87, 95% CI 1.80–4.59). In the multivariate model, predictors of ETI/death were age ≥ 75 years, the presence of a chronic ischemic heart disease, a respiratory rate ≥ 30 bpm after 24 h, and the development of suspected ARDS. In the multivariate model adjusted for HDW of admission, sex and NIRS used all significant variables confirming their predicted power.

**Table 4:** Univariate and multivariate analyses predict the probability of death or ETI

Variables	Univariate			Multivariate			Multivariate adjusted^a^		
	OR	95% CI	*p*-value	OR	95% CI	*p*-value	OR	95% CI	*p*-value
Age ≥ 75 years	4.63	2.91–7.37	< 0.001	8.69	3.6–20.7	< 0.001	4.74	2.28–9.84	< 0.001
Chronic ischemic heart disease, presence	2.63	1.47–4.72	0.001	2.88	1.09–7.61	0.033	2.76	1.15–6.6	0.022
Active cancer, presence	3.02	1.21–7.55	0.018						
Cerebrovascular disease, presence	2.59	1.24–5.41	0.011						
Kidney disease, presence	7.69	3.30–17.9	< 0.001						
*Evaluated at admission*									
SOFA score, +1 point	1.76	1.35–2.30	< 0.001						
PaO_2_/FiO_2_ ≤ 200	1.95	1.24–3.06	0.004						
Respiratory rate ≥ 30 bpm	1.94	1.10–3.39	0.020						
Hemoglobin ≤ 13 g/dL	1.83	1.18–2.83	0.007						
Procalcitonin ≥ 0.15 ng/mL	2.20	1.22–4.04	0.009						
D-dimer > 1000 ng/mL	2.60	1.57–4.32	< 0.001						
LHD > 300 U/L	2.12	1.17–3.82	0.013						
*Evaluated after 24 h of the NIRS use*									
PaO_2_/FiO_2_ ≤ 200	5.65	2.54–12.5	< 0.001						
Lactate + 1 mmol/L	1.65	1.07–2.55	0.023						
Respiratory rate ≥ 30 bpm	6.97	3.06–15.9	< 0.001	7.74	2.15–27.9	0.002	7.13	2.49–20.4	< 0.001
Heart rate ≥ 100 bpm	8.93	3–26.6	< 0.001						
NIRS, CPAP	1								
NIMV	2.87	1.80–4.59	< 0.001						
HFNC	1.46	0.61–3.53	0.394						
Shock, yes	3.54	1.35–9.29	0.010						
Suspected ARDS, yes	11.14	6.55–18.9	< 0.001	12.4	5.01–30.7	< 0.001	21.1	9.30–47.9	< 0.001
Acute ischemic heart complication, yes	8.95	1.09–73.6	0.041						
Acute kidney complication, yes	3.93	1.89–8.15	< 0.001						
Bacteremia, yes	3.65	2.06–6.45	< 0.001						
Acute neurological complication, yes	4.56	1.78–11.7	0.002						
Prophylactic LMWH, yes	0.46	0.29–0.72	0.001						
Therapeutic LMWH, yes	1.88	1.19–2.98	0.007						
Antibiotics, yes	2.69	1.22–5.92	0.014						

The Hosmer–Lemeshow test was *p* = 0.617 and *p* = 0.960 in the multivariate and multivariate-adjusted models

See Tables 1 and 2 for abbreviations. *OR* odds ratio, *CI* confidence interval, *NIRS* non-invasive respiratory support

^a^Model adjusted according to the internal medicine HDW of admission, sex of patients and NIRS used

The analysis comparing patients having or not at admission a DNI (or with no clear status) (Supplementary Information Table 2) showed a difference (in DNI) in the center of admission (more in Brescia), the age of patients (older) and the presence of comorbidities (arterial hypertension, atrial fibrillation, chronic ischemic heart disease, cerebrovascular disease, kidney disease, COPD more representative). Moreover, differences were evident in some variables evaluated at admission (DNI had higher values of the SOFA score, procalcitonin, and d-dimer while PaO_2_/FiO_2_ and hemoglobin were lower) and after 24 h (low PaO_2_/FiO_2_, high respiratory rate, and heart rate). The prevalence of patients having suspected ARDS (64%), such as in-hospital mortality (58%), was higher for DNI. Of note, there were no differences between using a specific NIRS and the DNI status.

Supplementary Information Figure 1 shows a flow diagram concerning the use of NIRS in DNI patients. The success of NIRS from admission appears higher in patients using CPAP (42%) as compared to NIMV (17%) and HFNC (9%). The treatment switch, with a similar trend to the total cohort (Fig. 1), was higher in CPAP patients (39% switch to NIMV), while 19% of patients using NIMV switch to CPAP during hospitalization; 82% of patients using at-admission HFNC switch to CPAP or NIMV. The failure of NIRS treatment (death) was very high in patients using NIMV (64%) as compared to CPAP (19%).

**Fig. 2:**
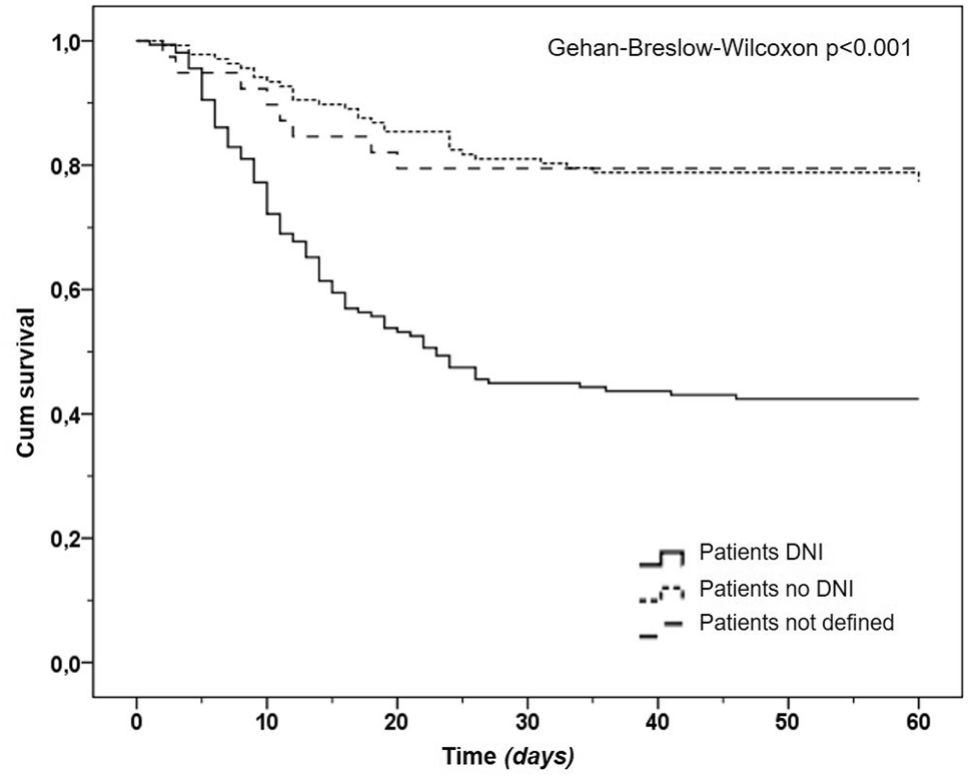
Kaplan–Meier curves according to the DNI status

In the Kaplan–Meier curves, the DNI status, compared to no DNI or patients without a precise definition, has a worse prognosis (Gehan–Breslow–Wilcoxon test *p* < 0.001, Fig. 2).

## Discussion

Our multicenter study that considered a real-life experience on patients with ARF due to COVID-19 pneumonia highlights two main findings. First, the use of NIRS in internal medicine HDW, newly organized to overcome the pandemic emergency, is feasible with reasonable effectiveness and second, although patients with DNI had a worse prognosis, there was a justifiable reason to treat them with NIRS. As compared to the available literature, the originality of this study lies in the multicenter experience that involved three internal medicine wards dedicated to NIRS. According to a recent meta-analysis, other non-ICU COVID units dedicated to NIRS were mainly composed by pneumologists or a by mixed personnel [8], while Internal Medicine Unit experiences were single center and involving a limited number of patients [8, 10].

### The new settings, the right candidate and the effectiveness of NIRS

The pandemic emergency and the dramatic shortage of hospital beds (especially in the first waves) have upset the ways to manage hospitalized patient with ARF. As a result, new management frontiers have been proposed, including high-intensity internal medicine wards [9]. In general, using NIRS outside the ICU has been demonstrated as a feasible option to cope with the massive demand for ventilatory assistance [5, 6, 8], with helmet CPAP being the most used approach [6]. In Italian experiences, COVID-19 internal medicine wards admitted patients with high clinical complexity and healthcare demand [11], demonstrating good efficacy in managing NIMV [10]. Similarly to what happened in most northern Italy hospitals, our three internal medicine units were rapidly converted into HDW dedicated to COVID-19 patients with ARF. Our patients were older than other reports on non-ICU settings from Italy [5, 6]. Moreover, multi-morbidity was highly prevalent with a high proportion of patients with DNI order application; of note, this last prevalence was the highest reported among those receiving NIRS outside of the ICU [5, 6, 10]. It was also higher in comparison to ARF patients undergoing NIRS in non-COVID settings, in which the pooled rate of DNI orders in studies from Europe was 28% [16]. Finally, concerning the severity of COVID-19 infection and the use of NIRS in an outside ICU setting, our cohort had better oxygenation at admission in comparison to other reports [5, 6, 10]). Similarly, our SOFA score was lower [5, 10]).

In a large cohort of COVID-19 patients, NIRS failure has been documented in 37 and 22% of patients, respectively [4]. A recent meta-analysis of case series outside the ICU reported an overall intra-hospital mortality of 36%, while NIRS failure was reported in 26% [8]. Mortality rates at 30 days using HFNC, CPAP, and NIMV outside ICU, were 16, 30, and 30%, respectively, while the corresponding ETI rates were 29, 25, and 28% [5]. Some technical characteristics related to pressures in our patients (see the PEEP and PS/PEEP for CPAP and NIMV groups, respectively) were slightly lower if compared to other cohorts [5]; this may be in line with the less lung impairment. We observed a significantly higher number of ETI/deaths in the NIMV group [5–7], compared to the CPAP group, in line with other retrospective studies suggesting CPAP as the preferred initial ventilatory strategy of ARF due to COVID-19 [17]. Our patients treated with NIMV were older with worse functional (PaO_2_/FiO_2_ and lactate values) [18] in which we noted some clinical aspects especially related to a possible bacterial co-infection (documented by leukocytes, neutrophils, procalcitonin, and prevalence of bacteremia) (Tables 1 and 3). In this context, the higher prevalence of patients using antibiotics and therapeutic LMWH in the NIMV group may be considered a coexistent pharmacological approaches used during hospitalization. With the exclusion of hydroxychloroquine (no patients in HFNC have used it), the pharmacological treatment contextual with the indication of the historical moment was similar among all patients with ARF. Considering CPAP as the reference, NIMV use was associated with a worse prognosis (Table 4). The prevalence of co-infections (21% of documented bacteremia) in our case series was in line with other reports (19% in [19]), also confirming the poor outcome of this subgroup [19]. As expected, the rate of switching to another NIRS was higher in the CPAP group compared to NIMV. Of note, we observed an unexpected phenomenon: a relatively high number of switches in the same patient (for example, patients initially receiving CPAP, then switched to NIMV, and finally again to CPAP), reflecting two specific aspects. First, the high degree of clinical variability during hospitalization, including the different compliance and degree of collaboration of multi-morbid elderly to NIRS modalities/interfaces, and second, our attempts to exploit any possibility of NIRS in the correct timing of disease, especially in the first two waves of the pandemic emergency when some pathophysiological aspects of COVID-19-associated acute respiratory distress syndrome were yet not known [20].

Regarding HFNC, the limited number of patients using this NIRS does not allow making meaningful inferences. Nevertheless, in our experience, most patients (83%) initially treated with HFNC were quickly shifted to another NIRS (median time to shift: one day). This was probably related in part to our few specialist competencies in this context, with difficulties in identifying the right candidate. Furthermore, a recent randomized controlled trial in patients with COVID-19 pneumonia and mild hypoxemia demonstrated that HFNC did not reduce the likelihood of escalation of respiratory support [21]. Interestingly, we noted that the early impact of NIMV or CPAP did not show significant gas analysis and clinical (respiratory and heart rate) changes to justify the severity of the condition not compensated by NIRS. Finally, it is also interesting to note that there were no differences between the DNI status and the required NIRS.

### Predictors of worse prognosis

Concerning predictors of death/ETI, our data confirm the importance of baseline clinical characteristics of hospitalized COVID-19 patients, such as age and specific comorbidities like chronic heart disease [9]. Similarly, the development of suspected ARDS [20] was associated with an increased risk of a worse prognosis. Of note, the persistence of respiratory distress after 24 h since NIRS is a known predictor of NIMV failure in other non-COVID-19 hypoxemic conditions [23].

### DNI patients

There is a vivid debate on the risks of delayed intubation in patients using the NIRS [4]. Notwithstanding the early intubation within the first 24 h of ICU admission in patients with COVID-19, pneumonia was found to be an independent protective risk factor for mortality [24], a meta-analysis of non-randomized cohort studies [25] suggests that intubation timing may not substantially affect the mortality and morbidity of COVID-19 patients, making a reasonable wait-and-see approach with NIRS to reduce the need of intubations, especially in the dramatic setting of ICU beds shortage. The proportion of DNI patients during the first pandemic waves has been reported to range from 23 to 50% [6, 8, 10, 12]. In our experience, the high proportion of the DNI group was likely mainly related to the older age and the burden of comorbidities typical of the internal medicine setting (Supplementary Information Table 2). As expected and confirmed in a recent systematic review and meta-analysis [12], the mortality rate in COVID-19 ARF patients was substantially higher in DNI versus non-DNI/unestablished status (Fig. 2). Although the intra-hospital overall mortality rate in our DNI patients was 58% (69% in those using NIMV), this was lower than that reported by two meta-analyses, the first considering more than 3 thousand patients requiring NIRS outside ICU (72%) [8] and the second considering more than one thousand five hundred of patients received DNI orders (84%) [12]. Thus, the success rate of NIRS in the DNI group could be considered appreciable, especially in patients using CPAP (47%). As shown in Supplementary Information Figure 1, the NIRS approach was overall similar in DNI versus non-DNI groups, including switching among different supports.

### Strength and limitations

This real-life experience in a large cohort of COVID-19 patients with ARF shows the feasibility of NIRS in a novel setting such as internal medicine HDW during the pandemic emergency. The high degree of switching between different types of NIRS may reflect the complexity and evolution of the COVID-19 clinical picture and the need to find alternatives in a novel and uncertain setting by devoted physicians rapidly engaged during the unexpected pandemic. The high proportion of DNI patients admitted to our internal medicine wards was unique, and the overall success rate of NIRS in this specific group without alternatives during the first two waves of the COVID-19 pandemic indicates the feasibility and worthiness of such approach. On the other hand, our study has major limitations including the retrospective data collection, the lack of data regarding close clinical monitoring after the first 24 h, preventing a more accurate evaluation of the true impact of NIRS on ARF. Similarly, we could not systematically collect imaging studies (e.g., lung CT scan) with missing features and accurate information for the definition of ARDS, as well as on the short-term evolution of COVID-19-related ARF during NIRS [26].

In conclusion, our retrospective study considering ARF patients with COVID-19 admitted to the new internal medicine HDW documents a good feasibility of the NIRS with an acceptable success rate. Although DNI patients had a worse prognosis, NIRS could be considered a reasonable chance of treatment.

## Supplementary Information

Below is the link to the electronic supplementary material.

Supplementary file1 (DOCX 265 KB)

## Data Availability

The data that support the findings of this study are available from the corresponding author [EC], upon reasonable request.
