# Supplementary material for: Use of non-invasive respiratory supports in high-intensity internal medicine setting during the first two waves of the COVID-19 pandemic emergency in Italy: a multicenter, real-life experience
Source: Intern Emerg Med. 2023 Jul 20;18(6):1777–87. doi: 10.1007/s11739-023-03371-z (PMC10504094; doi:10.1007/s11739-023-03371-z)

**Table 1 supplementary information.** Technical characteristics related to the first NIRS used.

| Variables | All patients | CPAP | NIMV | HFNC |
| --- | --- | --- | --- | --- |
| Interfaces |  |  |  |  |
| Oro-nasal mask, % | 16 | 16 | 21 | - |
| Total face mask, % | 17 | 5 | 35 | - |
| Helmet, % | 54 | 79 | 44 | - |
| Nasal cannulas, % | 13 | - | - | 100 |
| PS, cmH_2_O | - | - | 16 [14-18] | - |
| PEEP, cmH_2_O | - | 8 [5-8] | 8 [7-9] | - |
| High flow, L/m | - | - | - | 60 [47.5-60] |
| FiO_2_, % | - | 60 [50-60] | 70 [57-92] | 50 [40-60] |
| Time of treatment, days | 4 [2-8] | 3 [2-7] | 6 [3-9] * | 1 [1-3] ** §§ |

Data are shown as median [25°-75° percentiles] or number (percentages).

*Abbreviations:* NIRS define non-invasive respiratory support; CPAP, continuous positive airway pressure; NIMV, non-invasive mechanical ventilation; HFNC, high flow nasal cannula; PS, pressure support; PEEP, positive end-expiratory pressure; FiO_2_, the fraction of inspired oxygen.

* p<0.05 *vs* CPAP; ** p<0.001 *vs* CPAP; §§ p<0.001 *vs* NIMV.

**Table 2 supplementary information.** Characteristics of patients according to the DNI status defined at admission.

| Variables | Patients DNI  n = 158  (47%) | Patients no DNI  n = 137  (41%) | Patients not defined  n = 39  (12%) | p-value |
| --- | --- | --- | --- | --- |
| Hospital of admission, VR/BR/MI, n (%) | 48 (30)/78 (49)/32 (21) | 60 (44)/60 (44)/17 (12) | 3 (8)/33 (84)/3 (8) | <0.001 |
| NIRS, CPAP/NIMV/HFNC, n (%) | 88 (56)/59 (37)/11(7) | 68 (50)/61 (44)/8 (6) | 27 (69)/8 (21)/4 (10) | 0.104 |
| Age ≥ 75 years, n (%) | 107 (68) | 41 (30) | 16 (41) | <0.001 |
| Female, n (%) | 59 (37) | 39 (28) | 14 (36) | 0.259 |
| Arterial hypertension, n (%) | 107 (68) | 73 (53) | 26 (67) | 0.031 |
| Atrial fibrillation, n (%) | 29 (18) | 5 (4) | 2 (5) | <0.001 |
| Chronic ischemic heart disease, n (%) | 40 (25) | 15 (11) | 4 (10) | 0.002 |
| Cerebrovascular disease, n (%) | 25 (16) | 7 (5) | 3 (8) | 0.009 |
| Kidney disease, n (%) | 33 (21) | 8 (6) | 1 (3) | <0.001 |
| COPD, n (%) | 19 (12) | 4 (3) | 2 (5) | 0.010 |
| *Evaluated at admission* | | | | |
| SOFA ≥ 3 score, n (%) | 62 (56) | 29 (29) | 6 (37) | <0.001 |
| PaO_2_/FiO_2_ ≤ 200, n (%) | 70 (46) | 50 (38) | 9 (24) | 0.034 |
| Respiratory rate ≥ 30 bpm, n (%) | 36 (33) | 31 (31) | 8 (36) | 0.887 |
| Heart rate ≥ 100 bpm, n (%) | 46 (29) | 35 (26) | 8 (22) | 0.594 |
| Haemoglobin ≤ 13 g/dL, n (%) | 84 (53) | 47 (34) | 22 (56) | 0.002 |
| Procalcitonin ≥ 0.15 ng/mL, n (%) | 47 (63) | 32 (38) | 11 (58) | 0.005 |
| D-dimer > 1000 ng/mL, n (%) | 78 (67) | 56 (47) | 10 (37) | 0.001 |
| C-reactive protein > 150 mg/L, n (%) | 40 (26) | 39 (28) | 14 (36) | 0.481 |
| *Evaluated after 24 hours of the NIRS use* | | | | |
| PaO_2_/FiO_2_ ≤ 200, n (%) | 101 (85) | 104 (81) | 16 (61) | 0.023 |
| Respiratory rate ≥ 30 bpm, n (%) | 26 (20) | 8 (8) | 5 (16) | 0.032 |
| Heart rate ≥ 100 bpm, n (%) | 22 (16) | 3 (3) | 2 (5) | 0.002 |
| Suspected ARDS, n (%) | 101 (64) | 67 (49) | 14 (36) | 0.002 |
| Deaths, n (%) | 91 (58) | 31 (23) | 8 (20) | <0.001 |
| Time to death, days from admission § | 11 [7-17] | 17 [9-24] | 10.5 [4.25-16.50] | 0.047 |

Data are shown as median [25°-75° percentiles] or number (percentages). Percentages are calculated on non-missing data.

*Abbreviations:* see Tables 1 and 2; NIRS, non-invasive respiratory support.

§ Calculated on deaths.

**Figure 1 supplementary information.** Flow diagram concerning the use of NIRS on DNI patients only.

*Abbreviations:* CPAP define continuous positive airway pressure; NIMV, non-invasive mechanical ventilation; HFNC, high flow nasal cannula.


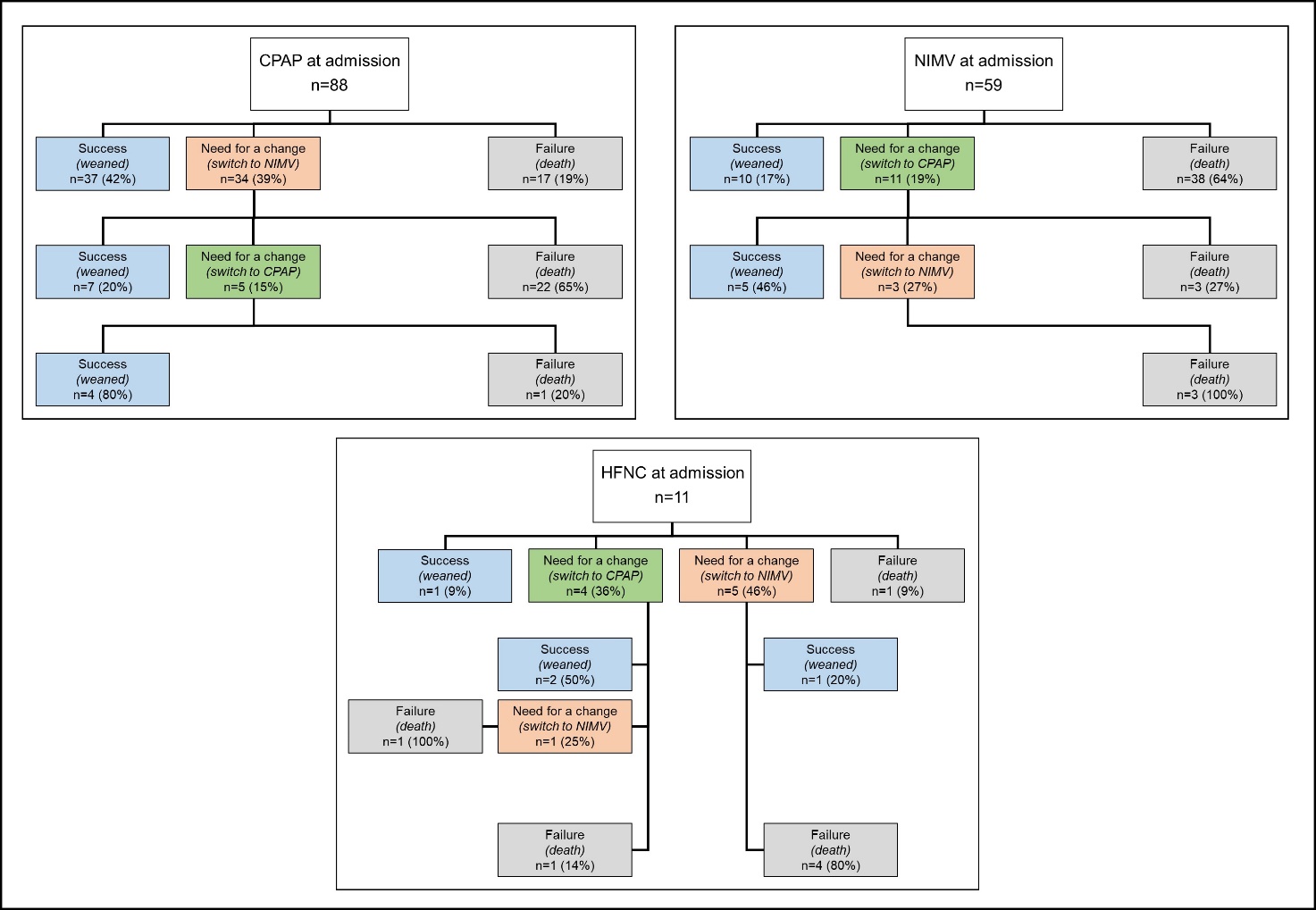

Supplement: Supplementary file 1 — Supplementary file1 (DOCX 265 KB) [file 11739_2023_3371_MOESM1_ESM.docx]
